# Hub-Centered Gene Network Reconstruction Using Automatic Relevance Determination

**DOI:** 10.1371/journal.pone.0035077

**Published:** 2012-05-03

**Authors:** Matthias Böck, Soichi Ogishima, Hiroshi Tanaka, Stefan Kramer, Lars Kaderali

**Affiliations:** 1 ViroQuant Research Group Modeling, University of Heidelberg, BioQuant BQ26, Heidelberg, Germany; 2 Institute of Informatics I12, Technische Universität München, Garching, Germany; 3 Department of Bioinformatics, Tokyo Medical and Dental University, Bunkyo-ku, Tokyo, Japan; 4 Institute of Informatics, Johannes Gutenberg-Universität Mainz, Mainz, Germany; 5 Institute of Medical Informatics and Biometry, Technische Universität Dresden, Dresden, Germany; Niels Bohr Institute, Denmark

## Abstract

Network inference deals with the reconstruction of biological networks from experimental data. A variety of different reverse engineering techniques are available; they differ in the underlying assumptions and mathematical models used. One common problem for all approaches stems from the complexity of the task, due to the combinatorial explosion of different network topologies for increasing network size. To handle this problem, constraints are frequently used, for example on the node degree, number of edges, or constraints on regulation functions between network components. We propose to exploit topological considerations in the inference of gene regulatory networks. Such systems are often controlled by a small number of hub genes, while most other genes have only limited influence on the network's dynamic. We model gene regulation using a Bayesian network with discrete, Boolean nodes. A hierarchical prior is employed to identify hub genes. The first layer of the prior is used to regularize weights on edges emanating from one specific node. A second prior on hyperparameters controls the magnitude of the former regularization for different nodes. The net effect is that central nodes tend to form in reconstructed networks. Network reconstruction is then performed by maximization of or sampling from the posterior distribution. We evaluate our approach on simulated and real experimental data, indicating that we can reconstruct main regulatory interactions from the data. We furthermore compare our approach to other state-of-the art methods, showing superior performance in identifying hubs. Using a large publicly available dataset of over 800 cell cycle regulated genes, we are able to identify several main hub genes. Our method may thus provide a valuable tool to identify interesting candidate genes for further study. Furthermore, the approach presented may stimulate further developments in regularization methods for network reconstruction from data.

## Introduction

With the development of large scale experimental platforms for the acquisition of genome-wide data, massive amounts of experimental data describing complex cellular processes are becoming widely available. The extraction of knowledge and development of models from such data remains a major challenge. Manual model development is constrained to small models involving a few dozen components, and requires extensive prior biological knowledge. The alternative is to use automated machine learning approaches to infer models directly from data, as reviewed by Kaderali and Radde [1].

For small models involving only a few dozen genes, detailed quantitative network inference approaches using *nonlinear differential equations* can be employed [2]. Such approaches fail for larger networks due to computational limitations and practical non-identifiability of model parameters. *Boolean network models* have been proposed as an alternative, neglecting the quantitative detail and assuming genes to be in only one of two states, active or inactive [3], [4]. Updates of the states are then done using logical rules, either synchronously for all genes or using asynchronous update rules [5]. Further extensions are based on fuzzy logic [6] or probabilistic Boolean networks, which basically use alternative sets of Boolean update rules that are stochastically employed [7].

*Bayesian networks* on the other hand are stochastic models that use conditional probabilities to describe dependencies between genes in a network [8]–[11]. These conditional distributions can be discrete or continuous, and are used to compute the likelihood of given data. Using Bayes' theorem, this is then used to compute the posterior distribution over alternative models given the data.

For large scale network inference involving thousands of genes, *relevance network approaches* are often used. They consider the similarity or dissimilarity between pairs of genes in a network, for example using pairwise correlation or mutual information, and use the “guilt by association” principle to reconstruct the underlying network. ARACNE is a representative approach of this type, it uses Gaussian kernel estimators to compute the mutual information between two genes, and then filters the resulting networks using different criteria [12].

Main challenges in automated network reconstruction arise from (1) The exponential growth of possible model topologies for increasing network size, (2) the high level of biological and experimental variability in measured data with often low signal to noise ratios, and (3) the frequently large number of different components that are measured, combined with an – in comparison – small number of different observations under changing conditions, e.g. number of time points or perturbations of the biological system. Together these problems lead to non-identifiability and overfitting of models. Regularization methods are therefore widely employed to penalize overly complex models.

The most commonly used regularization assumption in gene regulatory network reconstruction is that the inferred models should be sparse: There are typically only a low number of regulators acting on each gene [13]–[16]. Some studies furthermore indicate that the degree distribution in biological networks often follows a power law distribution, with only few highly-connected genes, and most genes having only a low number of interaction partners [17]. While there is ongoing debate about the statistical support of this claim [18], [19], it is widely believed that central hubs do exist in gene regulatory networks. This is usually incorporated into network inference approaches only indirectly, by limiting the number of regulators in the network [20], [21].

We here propose to use Bayesian networks with a Boolean state space to reconstruct transcriptional networks from gene expression time series data. We furthermore introduce a hierarchical prior distribution on the edge-weights in the network, which not only leads to sparse networks, but explicitly aims for the identification of central hub genes in the network, and centers the network reconstruction around these hubs.

We show results with the proposed approach on simulated as well as real experimental data sets of different sizes. Specifically, we present inference results on the genetic regulatory network controlling progression through the yeast cell cycle, based on three published genome-wide microarray studies. A first interesting result of our study indicates that large-scale network inference on this dataset is a very difficult problem, where none of the published methods we employed was able to significantly outperform random guessing. However, using the hierarchical prior presented in this work, key regulators could correctly be identified. Focusing our analysis on a smaller sub-network, we were able to reconstruct a core network regulating progression through the cell cycle. Our findings confirm that MCM1/SFF, CLB5/6 and CLN3 are key regulators in the yeast cell cycle network.

## Methods

### Network Model

We describe the activity of genes in a transcriptional network of 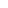 genes using discrete variables 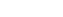, 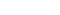, where 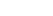 means that gene 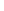 is active at time 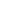, and 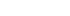 means the gene is inactive. We furthermore assume discrete time 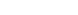, and model the time-invariant probability for each gene 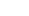 to be active at time 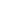, conditional on the states of all genes at the previous time point, 
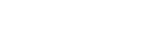
 using the probability distribution
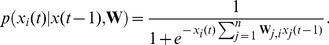
(1)

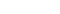
 is a weight matrix and describes the strength of regulation between all genes. In case of an activation of gene 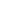 by gene 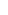, 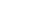, in case of an inhibition, 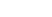, and 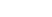 if there is no effect of gene 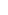 on gene 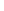.

Equation (1) describes a sigmoid function over the weighted sum of incoming regulations on a given gene 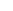. If the sum 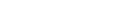 is positive, the probability that 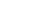 will be larger than the probability that 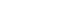, if the sum is negative, gene 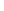 will more likely be inactive than active.

Summarizing the logarithm of the likelihood (1) over all genes and all time points, the log-likelihood of given data 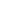 can be written as
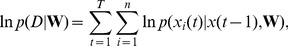
(2)where 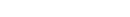 is the data, and 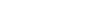 is the state vector of the system at time 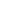.

We have previously used a similar model to reconstruct small signaling networks from RNAi perturbation data, see [22]. We are here extending this model for gene expression data, and use a hierarchical prior distribution to enable the hub-centered reconstruction of large-scale gene regulatory networks.

### Prior Distribution

For this purpose, we employ a hierarchical prior distribution on the regulation strengths 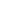 to regularize the network reconstruction. As first level prior, independent normal distributions with variance 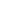 are used as prior on the weights 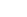, where the same variance 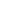 is used for all prior distributions over weights emanating from the same node 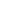:
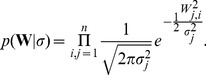
(3)The variance serves as hyperparameter, and determines the strength of the regulatory effect a given node 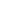 can have on all other nodes.

We furthermore use a second-level prior on the hyperparameter 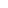. Since a standard deviation needs to be positive by definition, and should neither become too large nor too small, we use a gamma distribution on the 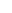, thus
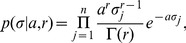
(4)with positive shape and rate parameters 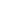 and 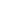, respectively, and gamma function 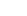.

Importantly now, the same value of 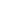 is used for all regulations exhibited by the same gene , i.e., for all *outgoing* edges for gene 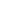. *Incoming* edges for a particular gene can have different values of 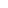. The combined effect of these two priors is that genes that receive a large weight also get a larger variance hyperparameter, and are more likely to attract further large edges in future inference steps, making the gene a hub. Correspondingly, genes with small weights get a small variance parameter 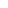, and it becomes increasingly difficult for these genes to attract large edges. Such a hub formation can not be achieved with ordinary sparseness priors such as L1 regression.

The shape and rate parameters 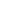 and 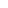 of the second-level prior ultimately control how large the weights of edges emanating from a particular node in the network can become. The choice of gamma distribution implies that most genes in the network have small variance hyperparameter 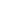. Only few genes receive large values of 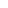, and hence, larger values for the weights on their outgoing edges. Pruning edges with small values would then directly lead to sparse networks, where edges are concentrated around central hub genes.

If we would allow different values of 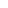 for each edge (i.e., 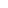 is a property of the edge, not of the gene), we would still obtain sparse networks where most edges have small values and only few edges receive large values, but the large edges would not center around hub genes anymore, but would be evenly distributed over the network.

We note that a similar automatic relevance determination (ARD) model has successfully been used in pattern recognition using neural networks by Neal [23], but the approach has not been used so far for genetic regulatory network reconstruction. Other related ARD approaches include Bayesian principal component analysis [24] and ARD-nonnegative matrix factorization [25].

The proper choice of prior hyperparameters (the shape and rate parameters 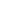 and 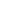) is critical to obtain optimal performance of the method. The values of 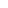 and 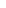 indirectly control how many hub genes there are. The regularization through the prior distribution should be sufficiently strong to learn hub genes and avoid overfitting, but regularization should not be too strong to completely dominate the learning from the data. “Good” values for 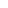 and 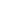 hence depend not only on the size of the network, but also the amount of experimental data available, the expected number of hubs in the data, and the level of noise in the data. The choice of parameters is hence a difficult issue, that – as with other Bayesian approaches and regularization parameters in general – requires a lot of experience and skill. We discuss this issue further at the end of the results section.

### Optimization of the Posterior Distribution

Given 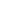, we can write the log-posterior distribution over 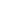 using Bayes' theorem as
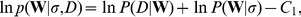
(5)where 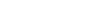 is independent of 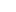 and can be neglected. Similarly, given 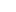, again using Bayes' rule, we can write the log-posterior distribution over 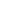 as
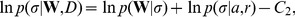
(6)where again 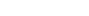 is independent of 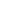 and can be neglected.

We now iteratively optimize equation (5) with respect to 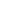 and equation (6) with respect to 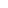, until the optimization converges. The idea here is that the optimization with respect to 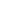 serves to reconstruct the network, whereas the optimization with respect to 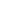 controls the magnitudes of the outgoing edge weights any given node 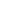 can have. If a node 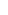 receives an outgoing weight with large value 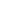, its hyperparameter 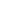 will increase in the next iteration, thus increasing the likelihood that other edges emanating from 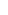 will also receive larger weights, making 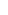 a hub gene. We note that the shape and rate parameters 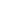 and 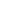 of the second level prior (4) indirectly control the expected number of hub genes.

The choice of starting point for optimization algorithms such as gradient descent is an important issue, depending on which different local or global optima can be identified in the optimization. We expect resulting networks to be sparse, and therefore, most of the weights 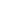 should be close to zero. We therefore suggest to start the gradient descent with respect to equation (5) at or in the vicinity of the origin, with a fairly large starting value of 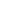 to initially avoid a strongly peaked prior distribution 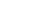.

The major disadvantage of gradient based optimization is that only a single maximum a posteriori estimate of 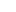 and 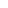 is returned. However, multiple different networks might explain given data, corresponding to different modes of the posterior distribution. Although we expect the resulting network to be sparse, starting the gradient descent at the origin for 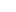 may results in getting stuck in a suboptimal local optimum. As an alternative for small networks, we therefore sample from the posterior distribution using the Hybrid Monte Carlo algorithm, a Markov chain Monte Carlo sampler that was originally proposed by Duane [26], see also Neal [23] and Kaderali [27]. The basic idea for our application is in each step of the Markov chain to randomly decide whether to sample from equation (5) or from equation (6) using hybrid Monte Carlo. These results can not only be used to validate the gradient based computations, but furthermore allow it to study the full posterior distribution over networks and model parameters, given the data. This is of particular value in case of multimodal distributions, when several different network topologies or sets of model parameters are consistent with the observed data.

### Evaluation of Networks

We use Receiver Operator Characteristic and Precision-Recall analysis to evaluate results of the network reconstruction. In our model, the 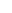 provide information on the importance of individual genes in the network, the 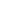 describe the inferred network topology. To assess the quality of reconstructed networks, we evaluated precision (fraction of true positives in all predicted regulations), sensitivity ( = recall, fraction of true positives in all actual positives) and specificity (fraction of true negatives in all actual negatives) of our approach. For this purpose, a variable threshold 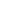 on the absolute value of the weights 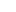 is introduced, edges with weights below the threshold are pruned from the network, and precision, sensitivity and specificity of edge recognition are then computed. Receiver Operator Characteristic (ROC) and Precision to Recall (PR) curves can then be plotted by varying the threshold 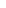 and plotting the resulting sensitivity over specificity, or precision over sensitivity (recall), respectively. Each value of 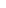 results in a specific point in these plots, the ROC and PR curves arise by varying 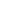 continuously and connecting the resulting points. ROC graphs nicely describe the overall relationship of positive to negative instances in the predicted model, and have the advantage to be insensitive to changes in the class distribution. On the other hand, precision to recall curves consider only the correctly inferred positive instances amongst all predicted links, and are therefore particularly useful for sparse networks. PR and ROC curves are then summarized further using the area under the curve (AUC), which is a value between 0 and 1. The closer this value is to one, the better is the reconstructed network. We compute the AUC for both ROC and PR curves.

We note here that the computation of sensitivity, specificity and precision usually requires two-class problems. In our context, three classes are possible for each edge – a positive regulation, an inhibition, or no regulation between two given genes. The assignment of predicted links to the four possible outcomes *true positive (TP), false positive (FP), true negative (TN) and false negative (FN)* used for the computation of sensitivity, specificity and precision is shown in Table 1. Importantly, the three classes imply that guessing a network will on average not result in an AUC value of 0.5 anymore, but values smaller than 0.5, depending on the number of activations, inhibitions and nonexistent edges in the true network. For a technical proof, see Mazur et al. [2].

**Table 1:** Evaluation of Predicted Networks.

	Predicted Regulation		
	Activation	Inhibition	No Regulation
**Actual Regulation**			
Activation	TP	FP	FN
Inhibition	FP	TP	FN
No Regulation	FP	FP	TN

Classifications of predicted links as true positives (TP), false positives (FP), true negatives (TN) and false negatives (FN). The assignment given here is used in the three-class classification problem to compute sensitivity, specificity and precision.

We performed a statistical test to assess the significance of the difference of the obtained AUC values from AUCs for randomly generated Networks. The null hypothesis is that the AUC of the ROC curve is not different from the AUC for guessing. Since our ROC curves are based on a three-class problem, we can not apply out of the box solutions for the calculation of the p-value. Therefore, we extended the methods of the R package pROC developed by Xavier *et al.* [28], which employs the method by DeLong *et al.* [29]. Briefly, the method of DeLong employs the mathematical equivalence of the AUC to the Mann-Whitney U-statistic. ROC curves can then be compared by evaluating the difference of the AUCs, which is asymptotically normal. To compare two AUC values, the method uses the covariance matrix for each of the ROC curves and finally does a two-sided t-test on the score of this comparison. To be be able to apply DeLong's method, we extended the Mann-Whitney kernel implementation of the pROC package as follows:
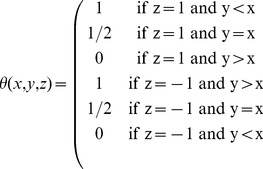
(7)with 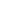 being the cases or TN, 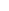 being the controls or TP and 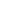 being the signs of the edges in the gold standard, either 1 or −1.

A further problem in the evaluation of reconstruction performance on real data arises due to the lack of a “gold standard” network. Hence, to evaluate the hub gene identification on real data, we extracted protein networks from the STRING database [30]. STRING calculates for each interaction a score based on the evidence from various sources like experiments, interaction databases or abstract text mining. It is clear that the PPI network reflects only a part of the gene regulatory processes but still, observations at this level can provide insight into the performance of the methods. STRING is also considering pairs derived from co-expression analysis and might therefore be more suitable than other PPI databases. We then computed the degree 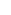 of each gene 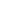 in the STRING network, and assessed correlations between 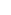 and the network inference hyperparameter 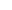. We then again used receiver operator characteristic analysis to study the predictive strength of 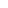 to identify hub genes, by varying a threshold on 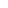 for a fixed threshold on the degree 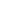, and computing sensitivity and specificity. ROC curves were summarized using the AUC, and AUC was plotted for continuously varied 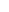.

## Results

We implemented our method in C++, using the gnu gcc compiler under the Linux operating system. All computations reported were carried out on a 3 GHz 64 bit Intel processor using a single processor core (no parallel processing). For a systematic evaluation of the approach, we used different simulated datasets, as well as real, publicly available microarray data.

Simulated data has the advantage that the real network underlying the data is known, and can be used to evaluate the performance of the network reconstruction and hub identification. We therefore discuss simulated data first. More specifically, we start by showing results using data that was simulated with the Boolean model used also in the inference method, using three different network sizes (11 genes, 100 genes, and 1000 genes), and using different dataset sizes generated from these networks for the inference task (20, 40 and 200 time points). This simulated dataset allows it to study the effect of network size and dataset size on performance of the network inference. To evaluate, whether the choice of prior introduces artifical hubs even on random networks where no hubs are present, we furthermore simulated data for a 1000 gene Erdös-Rényi [31] random network, again with different numbers of time points (20, 40 and 200 time points).

We next proceed by using a further simulated dataset, that was simulated with a realistic kinetic model for gene regulation, implemented in the GeneNetWeaver (GNW) package [32]. GNW uses systems of differential equations for simulation, data hence need to be discretized before they can be used in the network inference. GNW allows the simulation of time course data using a realistic model of noise for microarray data, this dataset hence allows it to study the effect of noise on the network reconstruction.

We finally applied our network inference method to three different publicly available microarray gene expression data sets regarding the yeast cell cycle, published by Spellman [33], Cho [34] and Pramila [35]. These three datasets were pooled, and network inference done on the ensemble dataset. We start by showing results on a small subset of the genes in this pooled dataset, representing a core network of 11 genes known to be involved in the yeast cell cycle. Thereafter, we present results on the reconstruction of a relatively large yeast transcriptional network comprising almost 800 genes. On this dataset, we compare results of our approach with results obtained using the relevance-network approaches ARACNE [12] and MRNet [36], as well as the Bayesian approach implemented in Banjo [37].

All analyses done and results achieved on simulated and real data are summarized in Tables 2 and 3.

**Table 2:** Overview of analyses on network inference.

Simulated Data									
Network	Nodes	Edges	TP	Gradient Descent			MCMC		
				ROC	p-val	PR	ROC	p-val	PR
**Boolean Model**									
Yeast Cell Cycle (CC)	11	34	20	0.74	0.0035	0.37	0.68	0.024	0.37
Core (Simulated)			40	0.76	0.0029	0.48	0.74	0.0045	0.48
			200	0.93	7.78e-08	0.67	0.91	6.4e-08	0.77
Mendes CenturySF	100	200	20	0.64	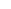 1e-8	0.13	0.43	0.0007	0.005
			40	0.75	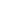 1e-8	0.3	0.52	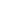 1e-8	0.04
			200	0.90	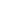 1e-8	0.66	0.67	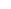 1e-8	0.19
Mendes JumboSF	1000	999	20	0.68	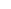 1e-8	0.05			
			40	0.77	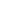 1e-8	0.26			
			200	0.88	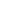 1e-8	0.62			
Random Network	1000	5000	20	0.42	-	0.003			
			40	0.62	-	0.09			
			200	0.79	-	0.4			
**GeneNetWeaver**					-				
No noise	100	532	25	0.53	-	0.053			
	250	1317	25	0.50	-	0.020			
	500	2150	25	0.50	-	0.008			
With noise	100	532	25	0.51	-	0.054			
	250	1317	25	0.50	-	0.021			
	500	2150	25	0.50	-	0.009			

Real Data									
Network	Nodes	Edges	TP	Gradient Descent			MCMC		
				ROC	p-val	PR	ROC	p-val	PR
Li et al. Yeast CC Core	11	34	98	0.56	-	0.27	0.59	-	0.26
Large Yeast CC Network	781	unk.	98	0.52	-	0.01			

Overview of all results on the simulated and biological datasets, using the approach presented in this manuscript. See the main text for comparison with other methods. Shown are results for the full network reconstruction task; table 3 shows corresponding results for hub identification. Each row in the table corresponds to one dataset. Nodes, edges and TP gives the number of genes, regulations and time points in the respective dataset. ROC and PR are the area under the curve values (AUC) of the Receiver Operator Characteristic (ROC) and Precision-Recall (PR) analysis, respectively. P-values were computed to test the null hypothesis of a significant deviation from random guessing for the AUC ROC values. Due to runtime limitations, MCMC results were calculated only for small networks, and p-values only for the synthetic networks with AUC values
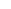
0.5. unk.: True number of edges for Yeast CC Network is unknown.

**Table 3:** Overview of analyses on hub identification.

Simulated Data					
Network	Nodes	Edges	TP	AUC Hub	
				Top 10 Hubs	Overall
**GeneNetWeaver**					
No noise	100	532	25	0.76	0.77
	250	1317	25	0.31	0.56
	500	2150	25	0.74	0.85
With noise	100	532	25	0.29	0.45
	250	1317	25	0.83	0.83
	500	2150	25	0.92	0.92

Real World Data					
Network	Nodes	Edges	TP	AUC Hub	
				Top 10 Hubs	Overall
Large Yeast CC Network	781	unk.	98	0.94	0.97

Overview of all hub identification results on the simulated and biological datasets. Hub AUCs were only calculated for the large networks since they are only of little relevance for small networks. Each row in the table corresponds to one dataset. Nodes, edges and TP gives the number of genes, regulations and time points in the respective dataset. AUC Hub is the AUC value computed for hub identification, shown are AUC values for the top 10 hub genes and maximum overall AUC values. A value of 0.5 corresponds to random guessing, values between 0.5 and 1 measure the hub identification performance. unk.: True number of edges for Yeast CC Network is unknown.

### Evaluation on Simulated Data

#### Simulation with Boolean Model

**Figure 1:**
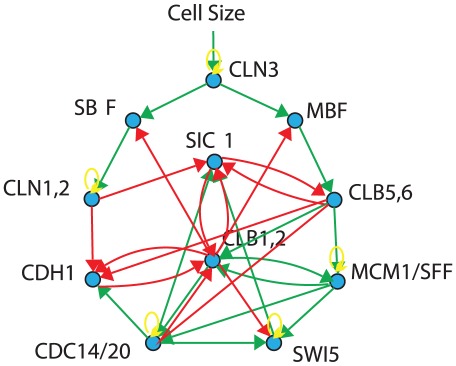
Yeast Cell Cycle Core Network. Core yeast cell cycle network, as derived by [33] from literature. There is one external checkpoint, cell size, which initiates progression through the cell cycle. Activations are shown in green, inhibitions in red, and self-regulations in yellow.

To systematically evaluate our network reconstruction approach, we simulated data for three different network topologies, with different numbers of genes. The smallest network contained 11 genes, and is the yeast cell cycle core network described by Li and coauthors [38], as shown in Figure 1. We furthermore used the *CenturySF* network topology comprising 100 genes, and the *JumboSF* network topology comprising 1000 genes, proposed by Mendes [39]. These topologies include desired properties such as regulatory loops, hub genes, and are sparse.

Weights for given topology were uniformly randomly generated between 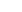 and 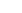, a starting state was randomly chosen, and time courses were simulated with 20, 40 and 200 time points, using the stochastic model described by equation (1). Weights in this range correspond to a moderate level of noise in the experimental data, due to the probabilistic model employed to simulate the data.

We then took the simulated data, and used our gradient descent and Markov chain approaches to reconstruct the underlying networks from the data alone. Shape and rate parameters of the gamma prior (4) were set to 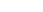 and 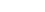 for these computations.

#### Results with Gradient Descent

Iterative gradient descent on the equations (5) and (6) was carried out as described in Methods, until convergence was reached. For the 11 gene network, computation finished in a few seconds. For 100 genes, computation time varied between 17 s for 20 time points, up to 4.1 min for 200 time points. On the 1000 gene network, gradient descent required 12.3 min for 20 time points, 90.5 min for the 40 time point data set, and 447.33 min or roughly 7 1/2 hours on the 200 time point data set.

**Figure 2:**
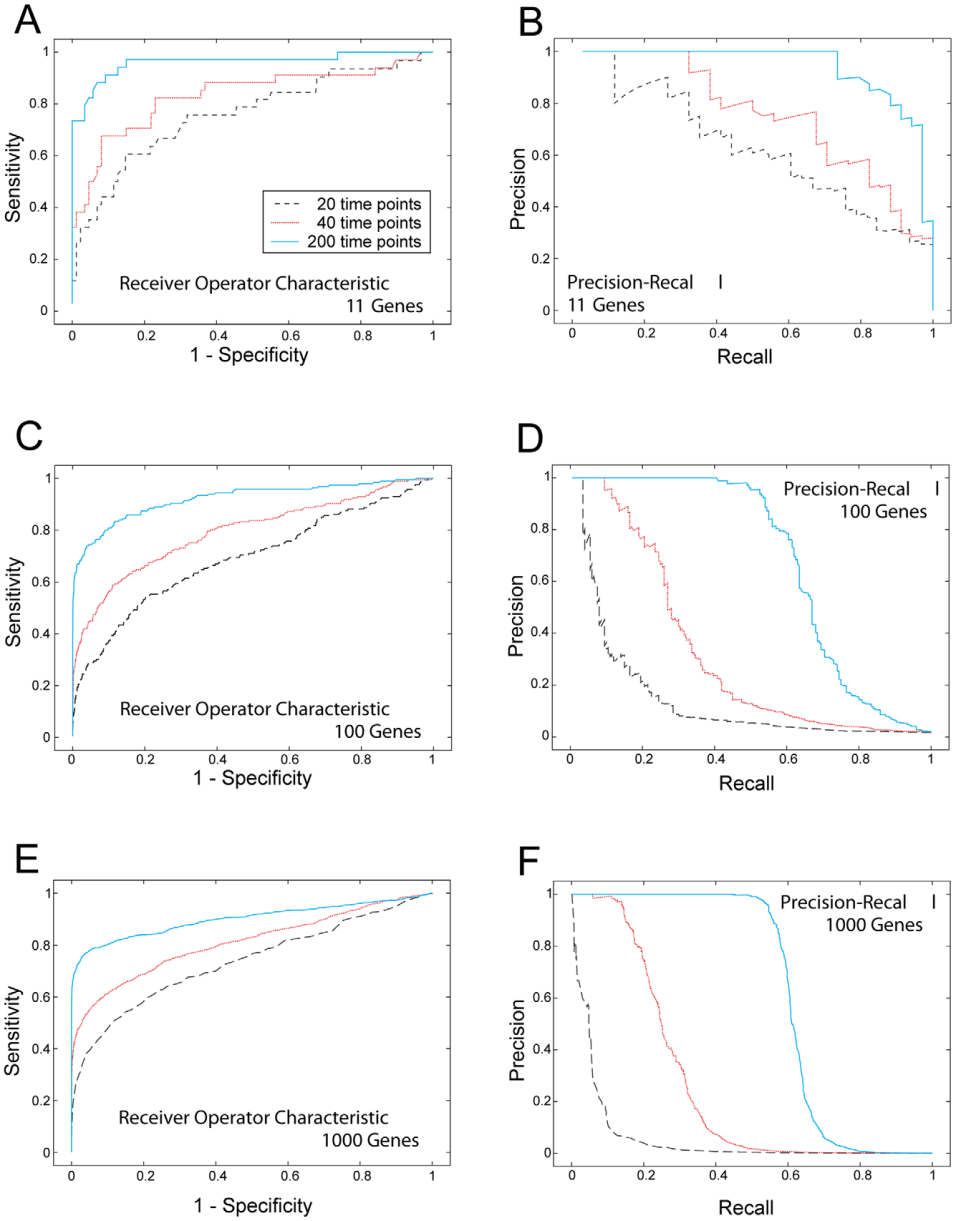
ROC and PR results, simulated data. The Figure shows receiver operator characteristic (ROC) and precision to recall curves (PR) for network reconstruction on simulated data, for different network sizes and different numbers of time points. A, B: ROC and PR curves for the network with 11 genes, C,D: ROC and PR curves for network with 100 genes, E.F: ROC and PR curves, respectively, for network with 1000 genes. Black: 20 time points used for network reconstruction, red: 40 time points, blue: 200 time points. It can clearly be seen how performance deteriorates with increasing network size and decreasing number of different time points. We note that, due to the three-class classification problem underlying the graphs, random guessing of network topologies would not yield a diagonal line in the ROC plots, but a significantly lower line with an area under the curve of approximately 0.33.

Figure 2 shows ROC and PR curves for the networks reconstructed from the data, in dependence of network size and number of time points available. As expected, for small network sizes (11 genes) and many (200) time points, the network reconstruction performs very well, and performance decreases with increasing network size and decreasing number of time points. Corresponding AUC values together with p-vales to assess the significance of the results (H0: AUC values are not superior to guessing, see methods for details) are shown in Table 2. To put these results further into perspective, we generated 1000 random “reconstructed” networks with 11, 100 and 1000 genes each, by drawing weights from a standard normal distribution, and computed the AUC for these networks. For the 11, 100 and 1000 gene networks this yields an average 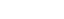 of 0.34, 0.38 and 0.39, respectively, and an average 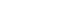 of 0.14, 0.009 and 0.0005. This reconfirms that results are significantly better than random.

#### Results with MCMC

We next repeated the computation using the Markov chain Monte Carlo sampling approach. Due to the high running time, an evaluation was done only for the 11 and 100 gene networks, by iteratively sampling from the distributions (5) and (6). 1 million sampling steps were done for the 11 gene network. Due to runtime constraints, only 800.000 steps were done on the 100 gene network. Running times for 20, 40 and 200 time points were 116, 207 and 929 minutes for the 11 gene network, and 10, 20 and 80 days for the 100 gene network, respectively. We note that computations were done using a single processor thread, and significant speed-ups can clearly be expected from parallelization of the sampler.

To simplify analysis of the reconstructed networks, we summarized the different values sampled for each parameter by the mean. This clearly is a crude intervention, and disregards much of the additional information contained in the distribution, for example, in case of a bimodal distribution. More sophisticated methods such as cluster analysis, and the consideration of higher order moments, can be used here. In spite of this simplification, results for the 11 gene network were completely equivalent to results for the gradient descent method (see Table 2), indicating that in this simulated example, only one set of parameters corresponding to one network topology is consistent with the experimental data, and is recovered using both gradient descent and Markov chain. Results of the 100 Gene Network obtained using the MCMC sampler were still significantly better than guessing, but inferior to results obtained from gradient descent, compare Table 2. This is likely due to multiple local optima of the posterior distribution. In this situation, averaging over multiple modes leads to an average result with low posterior probability, and thus suboptimal results. Furthermore, the number of sampling steps carried out (800.000) may not be sufficient to achieve adequate sampling from the stationary distribution, but this was a limiting factor due to runtime.

#### Results on a Non-Hub Network

To test our approach for biases towards inferring a scale-free structure also if no such structure is present in the gold standard network, we tested the gradient descent method on a random (Erdös-Rényi) 1000 gene network (generated with igraph [40]) with 5000 interactions. The data set size is the same as for the scale free networks, we simulated each of 20, 40 and 200 time points as described above. Network reconstruction was then done using the same settings as above, with conjugate gradient descent.

**Figure 3:**
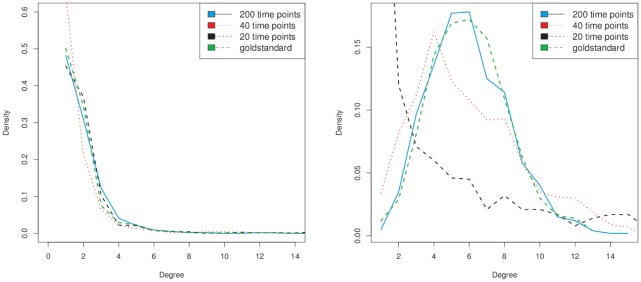
Inferred degree density distribution on scale-free and random networks. To test whether artificial hubs are generated in network inference due to their used prior distribution, we performed a comparative analysis on two different 1000 gene networks. The first network is the JumboSF network, a large scale-free network with central hub genes. The second network is a random Erdös-Rényi network, which does not contain any hubs. Network inference was performed using identical parameter values for the hyperparameters on both data sets. The figure shows the degree distribution of the inferred networks, in dependence of the number of time points used for network inference (left: JumboSF, right: random network). The plot shows that, provided sufficient data is available, the prior distribution does not lead to artificial hubs. On the other hand, if only little data is used for network inference, the prior starts dominating the results, as one would expect.

We focused our analysis of the results on the question if the network inference learns artificial hubs from the data, although none are present. Correspondingly, we evaluated the degree distribution of the reconstructed networks. Figure 3 shows the resulting degree distribution, for the 1000 gene scale free network above (JumboSF, Figure 3 left plot), as well as the Erdös-Rényi random network (Figure 3, right plot). The results clearly show that the approach does not identify artificial hubs, provided sufficient amounts of experimental data are available. In case of the data set with 20 time points, a distortion of the random network result can be seen. This data set is too sparse and the method cannot infer the right topology from it. In this situation the prior starts dominating the obtained results.

#### Simulation with GeneNetWeaver

As a further test of the method, we next simulated data using a realistic kinetic model, implemented in the GeneNetWeaver (GNW) package [32]. We subsampled networks of size 100, 250 and 500 from the Yeast transcriptional network implemented in GNW, and generated 25 time points using the ordinary differential equation model, with settings as for the DREAM challenge (see GeneNetWeaver documentation). Data were simulated without noise and with noise using the DREAM microarray noise model implemented in GNW. Data were discretized to Boolean states using a threshold of 50% on the maximum of the simulated gene activity levels. We then used the gradient descent approach with the hierarchical ARD prior presented, as well as a standard L1 sparseness prior to reconstruct the underlying networks from the data. Results of the network reconstruction were summarized by computing the area under the ROC curve for the reconstructed edges, as well as the area under the ROC curve for the hub identification.

Due to the low number of time points simulated, overall performance of the network reconstruction was not significantly superior to guessing in all runs. However, both the L1 prior as well as the hierarchical prior led to a successful identification of hub genes, and in all but one case superior performance of the hierarchical ARD prior. Results are summarized in Table 4. Interestingly, in case of the smallest network simulated, the addition of noise was so detrimental that no successful hub identification was feasible using either method. This probably reflects the situation that when more genes and hence more edges are present in a network, the influence of noise on the hub identification is less severe simply due to more edges contributing information on an individual hub gene. Overall, the results indicate that in the simulated data, information content seems not sufficient to reconstruct the full network, but it is still possible to identify key regulatory genes. Together, these observation motivate the use of hub-centered methods in particular on larger networks, where full reconstruction of a network is very difficult or even fails completely, but still some information on hubs can be extracted.

**Table 4:** AUC results for network reconstruction and hub identification on simulated data.

Network Size (Genes)	no noise		noise	
	L1	ARD	L1	ARD
	**Network Reconstruction**			
100	0.508	0.527	0.510	0.511
250	0.499	0.499	0.504	0.504
500	0.504	0.497	0.499	0.496
	**Hub Identification**			
100	0.526	0.767	0.449	0.453
250	0.789	0.563	0.755	0.827
500	0.698	0.849	0.859	0.924

Data was simulated using the GeneNetWeaver package, subsampling networks of size 100, 250 and 500 from the yeast transcriptional network. Simulation was done using an ordinary differential equation model, with and without experimental noise added to the data. Network reconstruction was carried out using the model described, using an L1 and a hierarchical automatic relevance determination (ARD) prior, respectively. Shown are the area under the ROC curve values for the correct identification of edges (top) and hub identification (bottom). A value of 0.5 is equivalent to guessing, a value of 1 corresponds to perfect identification of hub genes.

### Results using Microarray Data

#### Core Network of the Yeast Cell Cycle

We next evaluated our reverse engineering approach using publicly available microarray data regarding the yeast cell cycle. Data were pooled from the studies by Spellman [33], Cho [34] and Pramila [35]. We discarded the CDC15-synchronized data from the Spellman data set, due to previous reports of quality problems [41]. Experimental measurements were interpolated using smoothing splines, and binarized using the median of each gene as threshold. Missing values were interpolated with the mean of the preceding and the following time point. This discretization of the data into binary (Boolean) states can lead to several consecutive time points without any changes in all genes, such time points were then collapsed into a single time point, i.e. repetitive states after the binarization were removed. Network inference was performed using all time series simultaneously.

As reference network to evaluate the performance of our reconstruction, we used the 11 gene yeast cell cycle model proposed by Li et al. [38], see Figure 1. This network was carefully constructed from the literature, and we constrained our further analysis on reconstructing the interaction network between the 11 genes contained in this core network.

Network reconstruction was done using gradient descent, with shape parameter 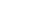 and rate parameter 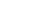. Precision, sensitivity and specificity for reconstructed networks were computed as described in methods, and used to plot receiver operator characteristic and precision to recall curves. The area under the curve was then calculated, resulting in 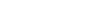 and 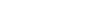. As it has been done for the synthetic networks, we generated 100 random networks and computed the AUC for these networks. This yields an average 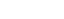 of 0.35 and an average 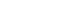 of 0.13, indicating that our approach performs significantly better than guessing.

**Figure 4:**
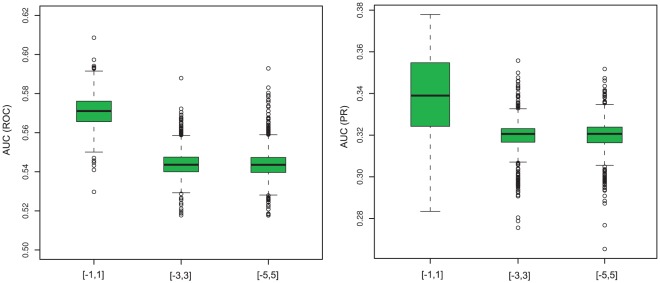
Effect of Starting Point on obtained AUC values. Shown are the distribution of AUC values (left: ROC, right: PR) of 1000 gradient descent runs, for randomly chosen starting values for 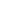, on the yeast core network. For the parameter vector 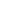, randomly chosen values within ranges of 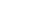, 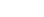 and 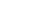 were used as a starting points for the calculations with CG. This was done for each of the suggested ranges 1000 times, and AUC ROC and AUC PR values were computed. The boxplots show the comparison between the different AUC values for these calculations. It can be clearly seen, that randomly sampled start values close to zero allow the approach to obtain better results for the optimal values of w. If the range of initial values for 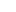 is too large, the optimization ends in suboptimal local optima corresponding to overly complex networks with many non-zero edges.

To furthermore study the effect of the choice of starting point for the gradient descent, we performed computations with different starting values, results are summarized in Figure 4. These results support the choice of the origin as starting point for the gradient descent, which seems to give good results. The rationale here is that we expect sparse networks, hence most edges should have weights equal to or close to zero. Apparently, if largely distinct values are chosen, the optimization tends to get stuck in local optima corresponding to overly complex, non-sparse networks.

We next repeated the network reconstruction using the Monte Carlo sampler, using 800.000 iterations and a burn-in phase of 50.000 steps. Computation time was 264 minutes, or 4 hours and 24 minutes. To check for convergence of the Markov Chains, several chains were run with different starting points, length, and random seed, and results were compared, indicating good convergence of the chains to the stationary distribution. We summarized values sampled for each model parameter by the mean, and used this to evaluate the reconstruction performance. Results overall were very similar to the ones obtained using gradient descent, with 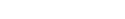 and 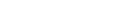, again significantly outperforming guessing.

Interestingly, obtained values for the hyperparameter 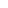 were very similar for all genes, both for the Markov chain Monte Carlo and the gradient descent approach. This probably reflects the fact that on such small networks, consisting of only 11 genes, the definition of hub genes is not or only marginally useful, and does not significantly influence network reconstruction. Still, largest hyperparameter values were attained by MCM1/SFF, CLB5/6, SBF and CLN3 which are key genes in the cell cycle network. For example, CLN3 initiates the cell cycle, or the transcription factor MCM1/SFF controls downstream genes like CLB2, CDC20 and SWI5.

**Figure 5:**
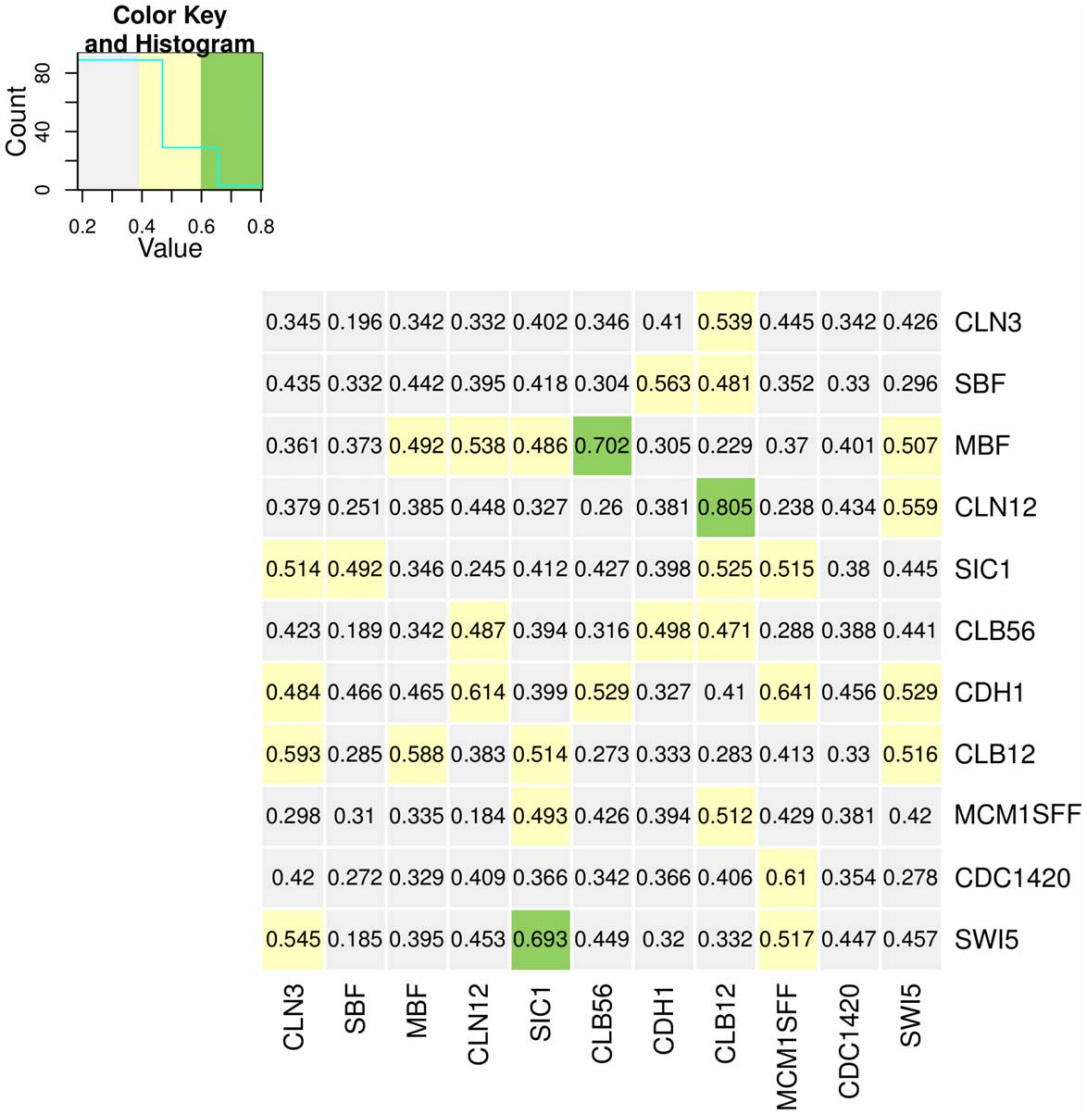
Multimodal Distributions in the Yeast Cell Cycle Core Network. Shown are Dip scores for the distribution of sampled edge weigths from the Markov chain. The Dip value measures the departure of an empirical distribution from the best fitting unimodal distribution. Large scores indicate a stronger deviation from unimodality. Rows in the diagram represent source (regulating) genes for edges, columns the target (regulated) genes. Colors have been used to indicate the magnitude of the deviation from unimodality.

It is clear that an analysis based on the mean of all values sampled for each parameter is a major simplification, and will actually yield inferior results in case of multimodal distributions. We have sampled 750.000 different values for each edge from the posterior distribution over model parameters, given the data, and clearly, this data can not only be used to provide confidence intervals on parameter estimates, but might also point to alternative topologies consistent with the data. To gain a better picture of the landscape of different modes and thus possible alternative topologies, we used the Dip test of unimodality on the Markov chains. This test, suggested by Hartigan and Hartigan (1985) [42], measures the departure of an empirical distribution from the best fitting unimodal distribution. The smaller this Dip-score statistic becomes, the more likely the distribution is unimodal. Due to the large sample size used in our Markov chains, the Dip test would reject the null hypothesis of unimodality for all edges in our network. We hence directly use the Dip value as a measure of the “deviation from unimodal”. Figure 5 shows the average Dip values for three prior settings (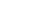 and 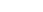, 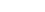 and 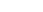, 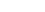 and 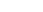), indicating that several of the edges show clear multimodal distributions. These edges could now be characterized further experimentally, to assess the true underlying network.

### Hub Genes in Yeast Transcriptional Regulation

The previous example on the core cell cycle network regards a relatively small network. For such small networks, the definition of hub genes is not so useful, and accordingly, the parameters 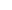 describing the importance of individual genes in the network were all similar, and essentially peaked at the mode of the prior distribution (4). To evaluate hub genes in larger networks, we took the set of 800 cell cycle regulated genes reported by Spellman et al. [33], and intersected this gene set with the genes in the Pramila data set [35], resulting in a set of 781 genes. Data were preprocessed as described above, network reconstruction was carried out using gradient descent. Shape and rate parameters of the prior were set to 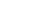 and 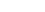, posterior optimization took 145 minutes. Computation with the Markov chain sampler is not feasible for this large network due to excessive running time. We furthermore used ARACNE, MRNet and Banjo for comparison, and furthermore repeated the computation with the model (2) using a standard L1 sparseness prior. ARACNE and MRNet results were computed using the R package minet [36]. ARACNE results were computed using default parameters in the minet implementation. Since minet uses additive tolerance instead of multiplicative tolerance, we furthermore used parmigene [43] to compute ARACNE results, and applied DPI thresholding at three different thresholds from 0.01, 0.05 and 0.15. MRNet results were computed using the Spearman estimator, the number of bins was set to 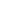 with 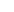 being the number of samples, as suggested in the documentation. Banjo was run with default parameters.

Since our simulation study indicates that at least 200 time points are required to successfully reconstruct a network of the given size, it is clear that individual edges predicted in our inferred network must be interpreted with great caution and need further experimental validation. In fact, we directly evaluated the reconstructed networks by comparison with the String database, using only experimentally verified or all interactions. We computed sensitivity/specificity and precision/recall of the reconstructed networks, and plotted ROC and precision-recall curves. None of the methods was able to perform better than guessing on this pooled dataset (Area under the curve for ROC [PR] analysis: Hierarchical Prior 0.515 [0.0106], L1-Prior 0.49744 [0.059], L2-Prior 0.496 [0.058], ARACNE 0.4993 [0.0099], Banjo 0.500 [0.06], MRNET 0.498 [0.059]).

**Figure 6:**
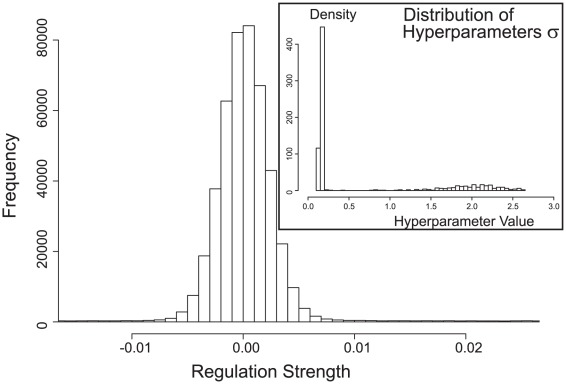
Hub Genes in the Yeast Cell Cycle. Histogram of reconstructed regulation strength for the full yeast cell cycle dataset. Negative weights correspond to inhibitions, positive weights to activations. Weights in the vicinity of zero indicate no regulation between two genes. The plot shows the distribution of regulation strengths between any two genes, showing clearly that only few genes exhibit strong regulations. The inset shows a histogram of the corresponding hyperparameters 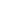 (equation 6), controlling the magnitude of the regulations exhibited by a particular gene. As can clearly be seen, most genes have only small importance corresponding to low values of 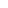, and only few genes are assigned large values of 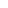 and correspondingly large weights on their outgoing connections.

We will therefore concentrate our analysis of this large reconstructed network on the identification of central hubs predicted. Figure 6 shows a histogram of the reconstructed regulation strengths for the 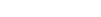 possible regulations between all pairs of the 781 genes. Negative weights correspond to inhibitions, positive weights to activations, and weights in the vicinity of zero indicate no regulation between two genes. The inset in the figure shows the distribution of hyperparameters 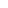 for the 781 genes, providing a direct measure of the importance of individual genes. A large value of 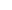 for a gene 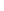 indicates that the gene has strong (positive or negative) effects on other genes. For example, 114 genes (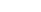) have a hyperparameter of 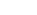 and 209 genes (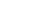) have 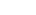, predicting that these genes play important roles in the yeast gene regulatory network.

Since predicted regulation strengths are continuous, we pruned all weights with absolute value 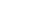 from the network. This yields a network with average out-degree 2.65. A plot of the correlation between the number of other genes regulated by a gene and 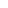 shows a good linear correlation (Pearson 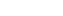, plot not shown), reconfirming that 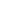 appropriately summarizes the genes importance in the reconstructed network. 114 genes have a hyperparameter value 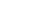, they on average are predicted to regulate 16.8 other genes, whereas an average gene in the full network regulates only 2.65 other genes.

We next evaluated in more detail genes identified as “hubs” in the transcriptional network. We retrieved interactions between the 781 genes in our dataset from the STRING database, using all interaction types. We then computed the degree 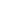 of each gene 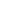 in the STRING network, and assessed correlations between 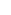 and the network inference hyperparameter 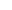.

Pearson correlation between 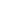 and 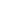 was only weak (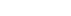), probably due to the large number of non-hub genes contributing significant noise to the correlation coefficient, and possibly also influenced by false positives in the database network. Accordingly, correlation improves to 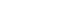 if the top 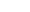, 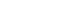 if the top 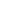, and 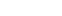 if only the top 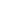 predicted hub genes are used.

**Figure 7:**
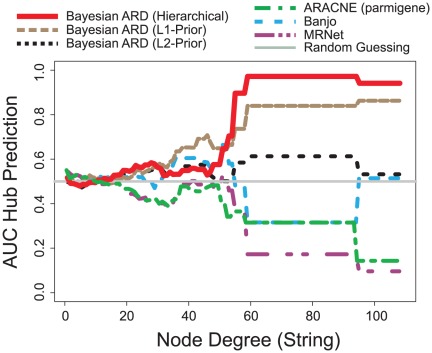
Receiver Operator Characteristic Analysis for the Prediction of Hub Genes in the Yeast Cell Cycle. Genes were split in two groups “hub” and “non-hub” based on a threshold 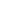 on the degree of the gene in the literature derived network, and ROC curves were computed by then varying the threshold on 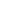. ROC curves were summarized for each 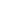 using the area under the curve. The plot shows 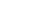 over 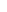. The red curve shows results for the inferred network using the method presented, the black dotted line shows results using the method with a Normal prior, the brown dashed line using a L1 “sparseness” prior distribution. The dashed blue line was obtained using Banjo, the dot-dashed green lines shows results of ARACNE, the dot-dashed pink line represents results of MRNet. The grey dashed line corresponds to the expected value for randomly guessing a network. Larger AUC values indicates better performance.

We then used receiver operator characteristic analysis to study the predictive strength of 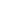 to identify hub genes, by varying a threshold on 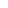 for a fixed threshold on the degree 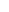, and computing sensitivity and specificity. ROC curves were summarized using the AUC, and AUC was plotted over different thresholds on the degree 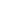, as shown in Figure 7. To compare results obtained using our approach with other methods, we reconstructed networks using ARACNE [12], MRNet [36] and Banjo [37], using the same input data. Importance values 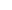 were then computed for each gene from the reconstructed edge weights as described above, and we then computed ROC and AUC values. We furthermore compared these results with a reconstruction using equation (2) with a normal and a L1 prior distribution, to study the effect of the hierarchical prior distribution used.

Figure 7 summarizes the AUC values obtained with these different approaches, in dependence of the STRING degree of the underlying genes. The dashed grey line in Figure 7 corresponds to the expected AUC for random guessing, the solid red curve shows the AUC for our ARD approach using the full posterior distribution. The dotted brown curve shows results using a L1 sparsity prior, the dotted black curve was obtained using a Normal distribution as prior. In comparison, the green and the pink dot-dashed curves were obtained using the relevance network approaches ARACNE and MRNet, respectively, whereas the dashed blue line shows results of the Bayesian method Banjo. While the Bayesian ARD approach performs only slightly better than guessing for low-degree genes (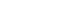), it makes excellent predictions for highly connected genes, which it identifies as hub-genes with high area under the ROC curve, and thus with high sensitivity and specificity. A comparison with the same model using an L1 and a normal prior shows clearly how the prior distribution used helps identify hub genes. Interestingly, at least on this dataset, the relevance network approaches ARACNE and MRNet performed worst, and actually make hub predictions that are inferior to guessing.

### Choice of Prior Hyperparameters

A critical issue is the choice of hyperparameter values 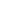 and 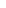 for the ARD prior. Optimal values for 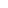 and 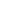 depend on the size of the network, the number of experimental data points, level of noise in the data, and expected number of hub genes. Some theoretical insight on the effect of changing 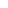 and 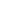 can be gained from a marginalization of the prior over 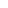:
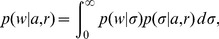
(8)which can be solved and analyzed numerically. By plotting 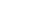 over 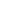 for different values of 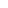 and 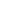, one can see that choosing smaller values of 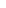 corresponds to a more “peaked” prior, i.e. a stronger “sparsity” of the inferred networks, whereas smaller values of 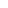 cause the overall importance of the prior to decrease. Hence, for larger networks and in case of small amounts of data, smaller values of 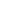 and larger values of 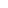 should be preferred, whereas in case of excellent and large amounts of data and small networks, 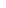 should be chosen larger and 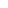 smaller, to decrease the influence of the prior distribution.

**Figure 8:**
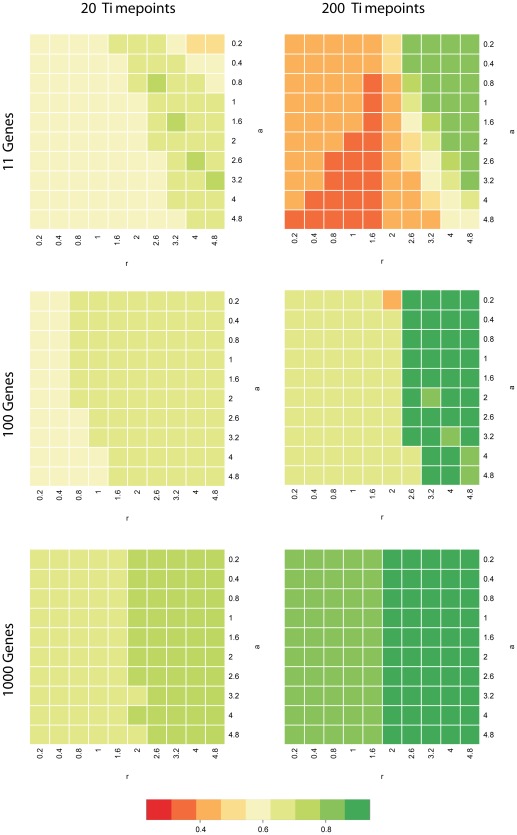
Sensitivity Analysis for the Network Inference performance on synthetic data with respect to parameters 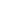
 **and** 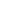 **.** Plots comparing distributions of AUC values for ROC graphs for different a and r settings (x- and y-axis), for the synthetic networks of sizes 11, 100 and 1000, using data sets with 20 and 200 time points, respectively. The plots show that results are relatively insensitive over a large range of parameters. Smaller values of the hyperparameter 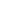 correspond to a more peaked prior distribution, resulting in “sparser” networks. Correspondingly, the figure shows that smaller values of 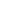 should be chosen for larger networks. Although the effect of changing 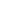 seems not as pronounced, larger values of 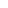 correspond to a narrower prior distribution, and should therefore be used if fewer data are available to avoid overfitting.

Due to the difficulty in manually choosing these parameters, we performed a sensitivity analysis to assess the sensitivity of results with respect to choices for 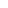 and 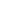. On the simulated data (synthetic 11 gene, 100 gene and 1000 gene data sets), we modified parameters 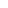 and 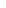 in the range from 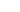 to 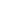, performed the network inference for each combination using gradient descent using 20 and 200 time points from the data, and computed resulting 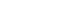 values. Results are shown in the heatmaps in Figure 8. The plots show that results are relatively insensitive over a large range of parameters. Smaller values of the hyperparameter 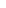 correspond to a more peaked prior distribution, resulting in “sparser” networks. Correspondingly, the figure shows that smaller values of 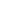 should be chosen for larger networks. In comparison, the correct choice of 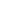 seems less important.

**Figure 9:**
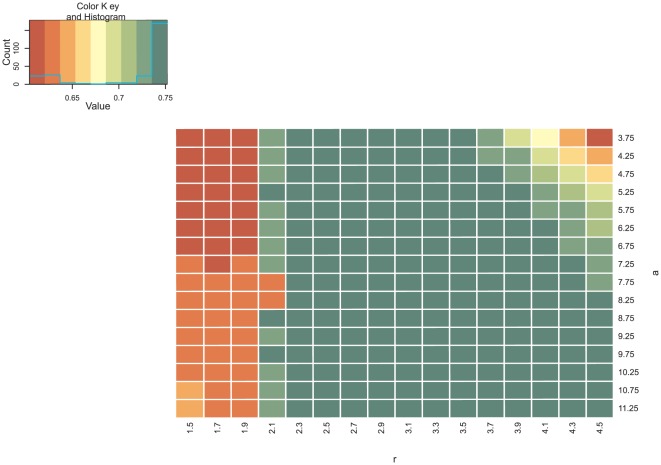
Sensitivity Analysis for the Prediction of Hub Genes in the Yeast Cell Cycle with respect to parameters 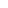
 **and** 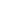 **.** To assess the effect of changes of model parameters 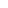 and 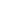, both parameters were varied individually and together by up to 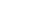 percent. Network reconstruction was restarted for each combination of values for 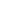 and 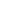, and average AUC values were computed for the reconstructed networks in comparison to the STRING network. The figure shows the resulting AUC values over 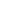, indicating that results are relatively insensitive over a wide range of parameter values.

On the experimental data regarding the hub genes in the yeast cell cycle, we also performed a similar analysis. We note that parameters chosen for this analysis (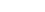, 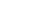) result in a significantly narrower distribution of 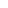 than the hyperparameter values used on the synthetic data, corresponding to much stronger regularization – in line with expected larger levels of noise in the data. We modified both parameters individually and together by up to 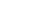, reran the network inference, and computed average AUC values for the reconstructed networks. Figure 9 shows the resulting AUC values, and clearly shows that in spite of considerable variation of the hyperparameter values over a wide range, performance is again only marginally affected.

## Discussion

In this paper, we present a novel approach to reconstruct gene regulatory networks from microarray gene expression time series data, which employs the concept of *hub genes* for regularization. Our evaluation on the simulated data shows that the method precisely retrieves the original network from the data, provided sufficient time points are available. Furthermore, the approach can help identify hub genes in regulatory networks, and we have shown an application to a large biological dataset regarding yeast, where we successfully identified several important hub genes.

While a considerable number of approaches to reconstruct networks from data have been published to date, to our knowledge, this is the first method that simultaneously identifies hubs in the regulatory network and centers the network reconstruction around these hub genes, by using a hierarchical Bayesian prior distribution on the edge weights. While clearly other network reconstruction approaches can also be used to identify hubs *in retrospect*, our approach specifically centers the reconstruction of the network around central hub genes. In particular on large, noisy datasets, this may be a major advantage over other approaches that may only identify clusters of correlated genes, but not necessarily induce a hierarchical structure. This is shown on the yeast cell cycle network, where ARACNE, Banjo and MRNet all failed to correctly identify the highest-degree hubs in the network. We therefore believe our approach to have high potential for the identification of hubs in unknown regulatory networks, around which further experimental effort should be centered in elucidating the respective network. Ultimately, this could be highly useful for an iterative procedure of network reconstruction, experiment design, further biological experiments, and feeding the results back into network reconstruction, of particular interest for large networks. Indeed, if certain hubs in a network are already known, this can even be integrated into the network inference by choosing a different prior over 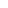 for the known hub genes.

We have shown two different approaches to evaluate the posterior distribution over models given the data. On the one hand, we used a Markov chain Monte Carlo approach to sample from the posterior distribution. The advantage of this is that full distributions are evaluated, hinting to possible different, alternative network topologies, yielding additional information on confidence in results. The disadvantage of this method is the computational burden involved, making it infeasible for networks involving more than a few dozen genes, at least without further parallelization of the sampler. On the other hand, we use gradient based optimization to maximize the posterior, yielding a single optimal network topology. This can be computed considerably faster and is feasible for networks with several hundred to thousands of genes, but does not provide any information on alternative, high-probability networks, and no confidence intervals are available on model parameters.

We showed results on simulated data, indicating that even with only moderate noise, for a network of approximately 1000 genes, at least 200 time points are needed for reliable network reconstruction. Hence, while the size of the used yeast data set clearly is not sufficient for a precise reconstruction of the whole network structure, we could identify important hubs in the regulatory network, which were validated using the STRING database. An interesting result from our point of view is that all published approaches that we tried, including our own, failed to reconstruct a yeast transcriptional network from the microarray data, at least in comparison to the gold standard network from the STRING database. This may be due to low quality of the experimental data and the lack of targeted interventions, but these results are in line with findings in recent results of the DREAM competition, where also even the best submitted methods showed surprisingly weak performance, and most methods did not perform better than guessing [44], [45]. Under conditions of high noise and limited amounts of experimental data, for large scale network reconstruction, a method that centers on hubs may therefore be of value to concentrate further experimental efforts and network reconstruction attempts around these hub genes.

Interestingly, the HUB prediction for the yeast dataset shows good performance with high AUC values if a fairly strict definition of hub genes is enforced, by requiring a hub to have a large number (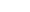) of interaction partners in the STRING network. If this threshold is relaxed, AUC values drop rapidly. We offer two explanations for this behavior: On the one hand, genes with low connectivity in the network probably contribute significant noise to the network reconstruction, simply due to their large number. On the other hand, false positives in the STRING dataset will affect genes with few interaction partners more than genes with a large number of partners, since a gene with say 100 interaction partners would still be considered a hub, even if 20 of the interactions are false. It is somewhat surprising that the transition occurs so rapidly around a value of 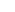 interaction partners, one would expect a more smooth transition where AUC gradually increases with increasing degree. To study this further and exclude the possibility that this is an artifact of the method employed, we additionally performed the same computation on the 500 gene simulated network with noise from GeneNetWeaver, where indeed a smooth increase of the AUC values is observed. We therefore speculate that the rapid transition in the Yeast dataset is not due to the method we used, but rather an artifact of the data set.

A difficulty in using our approach, that all Bayesian methods share, is the need to select parameters for the prior distributions. In some cases, these can significantly influence results, and the choice of parameters 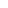 and 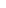 in our method is not straightforward. Optimal values depend on the size of the network, the amount of available experimental data, the level of noise in the data, and the expected number of hub genes. Importantly, our sensitivity analysis of the yeast cell cycle network reconstruction with respect to parameters 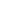 and 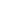 shows that results are relatively insensitive over a wide range of parameter choices. Still, considerable experience is required in tuning these parameters. Methods to assist finding sensible choices, such as empirical Bayes approaches or careful cross-validation, could be used to address these issues.

The main assumption we make in our model is the binarization of state space – each gene is assumed to be either active or inactive. This implies a loss of detailed expression levels, but allows us to tremendously reduce model complexity and computation time, and hence, to explore biological networks of a much larger scale. This discretization of the data may furthermore have advantages in case of microarray data as used in this study, in particular if the data is more of a qualitative than of a quantitative nature due to inherent noise, or if data from different platforms or different studies shall be integrated. Furthermore, in contrast to co-expression based approaches, the underlying Boolean model allows causative inferences, hence edges between genes are directed and can be interpreted not only as correlation or co-expression, but causality.

A difficulty associated with the use of a Boolean model is the requirement to discretize the experimental data. We have used smoothing cubic splines in this work to smooth out smaller fluctuations in the experimental data, and thus take care of some of the noise in the data. Data were then discretized for each gene separately by using the median of the respective gene as threshold. For the small 11 gene network, we have manually checked the resulting data, and the discretized values were compared with the raw data to assure that the interpolation and discretization has produced reasonable results. However, this is clearly not feasible for large scale network inference with hundreds to thousands of genes, and discretization can then become a difficult issue, in particular since it will clearly have a considerable effect on results of the network inference. Already using the mean instead of the median as discretization threshold can lead to a completely different data set, if the time course for a particular gene has a single large outlier.

The spline interpolation itself requires the choice of a smoothing factor, and clearly, also other interpolation functions could be employed (for example linear, polynomial, etc.). We have previously proposed an iterative procedure between spline interpolation and network inference for a model using ordinary differential equations [2]. In this work, model predictions are fed back into the interpolation, to adaptively choose parameters for the interpolation. It is not immediately evident how such a procedure can be used with a Boolean model, but this might be an interesting question for future work.

Overall, our results show that the approach presented may be a valuable tool for large-scale network reconstruction, and may guide experimental efforts to characterize identified hubs in more detail. The Boolean discretization used in principle allows the reconstruction of larger networks and may in fact be an advantage in case of noisy data, but our results also clearly indicate that an accurate reconstruction of a large network is not feasible with present limited data sets containing at most a few dozen time points or different conditions. In addition to larger experimental data sets, a key to overcome these challenges will be the integration of as much biological knowledge as is available. Our method contributes to this aim by providing a general framework for reconstructing sparse networks with small world properties.
